# Sevoflurane sedation in COVID-19 acute respiratory distress syndrome: an observational study with a propensity score matching model

**DOI:** 10.3389/fmed.2023.1267691

**Published:** 2023-09-15

**Authors:** Guglielmo Consales, Iacopo Cappellini, Benedetta Freschi, Laura Campiglia, Maddalena Parise, Lucia Zamidei

**Affiliations:** ^1^Department of Critical Care, Section of Anesthesiology and Critical Care Azienda USL Toscana Centro, Ospedale Santo Stefano, Prato, Italy; ^2^Anesthesia Unit, Castellanza Hospital, Multimedica Group, Milan, Italy

**Keywords:** sevoflurane, sedation, COVID-19, acute respiratory distress syndrome (ARDS), inhalatory sedation

## Abstract

**Introduction:**

The management of severe COVID-19-induced acute respiratory distress syndrome (C-ARDS) often involves deep sedation. This study evaluated the efficacy of sevoflurane, a volatile anesthetic, as an alternative to traditional intravenous sedation in this patient population.

**Methods:**

This single-center, retrospective cohort study enrolled 112 patients with C-ARDS requiring invasive mechanical ventilation. A propensity score matching model was utilized to pair 56 patients receiving sevoflurane sedation with 56 patients receiving intravenous sedation. The primary outcome was mortality, with secondary outcomes being changes in oxygenation (PaO2/FiO2 ratio), pulmonary compliance, and levels of D-Dimer, CRP, and creatinine.

**Results:**

The use of sevoflurane was associated with a statistically significant reduction in mortality (OR 0.40, 95% CI 0.18–0.87, beta = −0.9, *p* = 0.02). In terms of secondary outcomes, an increase in the PaO2/FiO2 ratio and pulmonary static compliance was observed, although the results were not statistically significant. No significant differences were noted in the levels of D-Dimer, CRP, and creatinine between the two groups.

**Conclusion:**

Our findings suggest an association between the use of sevoflurane and improved outcomes in C-ARDS patients requiring invasive mechanical ventilation. However, due to the single-center, retrospective design of the study, caution should be taken in interpreting these results, and further research is needed to corroborate these findings. The study offers promising insights into potential alternative sedation strategies in the management of severe C-ARDS.

## Introduction

In the initial phase of the COVID-19 pandemic, the need for alternative sedation methods became apparent (1). The utilization of deep sedation was observed in the management of these patients, facilitating aggressive ventilatory strategies (2). Furthermore, the risk of healthcare workers being exposed to SARS-CoV-2 due to patient agitation and self-extubation, especially during prone positioning, raised concerns. This surge in demand for sedative drugs resulted in significant global shortages (3).

Volatile anesthetics (VAs), particularly halogenated ethers, have been a fundamental part of general anesthesia for many years. They have also been recognized as a feasible alternative to intravenous sedation in intensive care units (4). Among these, sevoflurane is notable for its quick sedation induction, reduced irritative effect during administration, and beneficial bronchodilatory effect (5–8). Inhalatory sedation for critically ill patients is generally achievable at doses approximately one-third of those required for general anesthesia (9). However, the application of VAs in intensive care units poses challenges due to their delivery method and the risk of environmental contamination and proper gas disposal.

The introduction of devices such as Sedaconda ACD^®^ (previously Ana-ConDa^®^) (Sedana Medical, Danderyd, Sweden) and MIRUS (Pall Medical, Dreieich, Germany) has enabled the safe delivery of VAs in intensive care units, as they are highly adaptable to different types of ventilators used in these settings (10). Emerging evidence suggests that inhalation agents like sevoflurane may offer more than just sedation and could be beneficial for patients with COVID-19 related acute respiratory distress syndrome (C-ARDS) (11). These agents may provide anti-inflammatory capabilities, dose-dependent bronchodilation, and pulmonary vasodilation, which could lead to slight improvements in patient oxygenation (12–14).

Despite these potential benefits, current results do not conclusively demonstrate a decrease in mortality or a reduction in ICU stay length (15). However, inhaled sedative regimens have shown modest benefits in faster extubation times after drug discontinuation, which could potentially prevent ventilation-associated complications and provide a survival benefit (16).

In this study, our primary hypothesis was that the administration of sevoflurane to C-ARDS patients would lead to improved clinical outcomes, notably a reduction in mortality. Secondary outcomes were chosen based on their relevance to C-ARDS pathophysiology. Specifically, D-dimer was included as an indicator of prognosis in SARS-CoV-2 infection and to assess coagulation activity. CRP was chosen as a marker of systemic inflammation, and creatinine was selected to evaluate renal function.

## Materials and methods

This study was a retrospective, single-center, cohort study conducted at the COVID Intensive Care Unit of Santo Stefano Hospital in Prato, Italy, from March 2020 to June 2021. The study focused on patients with COVID-19 related acute respiratory distress syndrome (C-ARDS).

### Inclusion criteria

Patients had to provide informed consent.
Patients aged 18 years or older.
Confirmed COVID-19 case as indicated by a positive SARS-CoV-2 swab.
Patients diagnosed with ARDS as per the Berlin criteria.
Patients undergoing invasive mechanical ventilation.

### Exclusion criteria

Patients who did not provide informed consent.
Cases where ARDS was not related to Sars-Cov-2 infection.
Patients not on invasive mechanical ventilation.

### Cohort description

Patients were categorized into two groups based on the sedation received:

Group 1 (SEVO): This group consisted of patients who were sedated with sevoflurane within the first 72 h after endotracheal intubation. A minimum dosage amount was set for those who received at least 24 h of inhalatory sedation.
Group 2 (IV): This group comprised patients sedated with intravenous drugs, such as propofol and midazolam.

This format removes the bullets and organizes the information more clearly and in line with the reviewer’s feedback. If this is satisfactory, you can replace the relevant section in your manuscript with the reformatted content. During the surge of the pandemic, the healthcare system grappled with unprecedented challenges, one of which was the acute shortage of sedative drugs essential for the management of critically ill patients. Given this backdrop, the selection between inhaled and intravenous sedation wasn’t merely a clinical preference but a necessity-driven decision. Inhaled sedation, particularly using sevoflurane, became a crucial alternative. The choice of sevoflurane was influenced not only by its availability but also by its known safety profile and efficacy in providing adequate sedation. This strategic shift in sedation methodology underscores the adaptability of healthcare protocols in response to crisis situations.

In our effort to compare the two groups, we systematically extracted key demographic, clinical, and biochemical information from each patient’s medical record. This included age, sex, and body mass index (BMI), along with details of their smoking habits. We also considered any chronic conditions that were present upon hospital admission, such as chronic obstructive pulmonary disease (COPD), hypertension, diabetes, ischemic heart disease, and chronic renal failure.

Moreover, we recorded specific time-related parameters such as the time between symptom onset and hospital admission, the duration of non-invasive ventilation (NIV) before intubation, the duration of invasive mechanical ventilation, length of ICU stay, and the overall duration of the hospital stay.

To capture the progression of the disease and the patient’s response to treatment, we identified three critical timepoints during hospitalization:

T1, defined as the moment of intubation and initiation of mechanical ventilation.
T2, occurring 72 h after T1.
T3, marking 7 days after T1.

At each of these timepoints, we collected a set of biochemical parameters including Procalcitonin (PCT), C-reactive protein (CRP), Aspartate aminotransferase (AST), Alanine aminotransferase (ALT), D-Dimer, Lactate dehydrogenase (LDH), Creatinine, Blood urea nitrogen (BUN), and Creatinine Kinase (CK).

**FIGURE 1:**
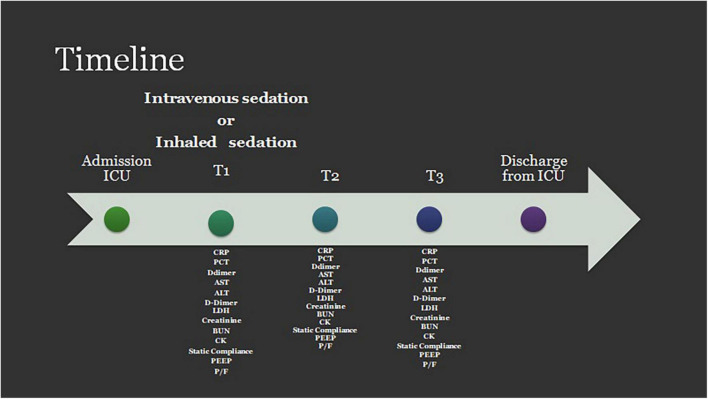
Timeline for data collection. PCT, procalcitonin; CRP, c-reactive protein; AST, aspartate aminotransferase; ALT, alanine aminotransferase; D, Dimer; LDH, lactate dehydrogenase Creatinine; BUN, blood urea nitrogen; CK, creatinine kinase; PEEP, positive end expiratory pressure; P/F, PaO2/Fio2 ratio.

To gain insight into the patient’s respiratory status and the mechanical characteristics of their respiratory system, we also collected the following respiratory and ventilatory parameters: the PaO2/FiO2 ratio, pulmonary static compliance, and the level of positive end-expiratory pressure (PEEP) (Figure 1).

The depth of sedation was assessed using a dual approach. It should be noted that this approach reflects the hospital’s strategy at the time, which aimed for deep sedation in all COVID ARDS patients. A Bispectral Index (BIS) device (Bispectral Index™ (BIS™) Monitoring System, Covidien, Dublin, Ireland) was employed, targeting a BIS Index of 40–50, a range typically associated with deep sedation and commonly used in intensive care settings to ensure patient comfort and safety during invasive procedures. Concurrently, the Richmond Agitation-Sedation Scale (RASS) was utilized, aiming for a score of −4/−5, indicative of deep sedation or an unarousable state. These measures, while correlated, provide different types of information: BIS offers an objective, physiological measure derived from EEG data, while RASS provides a subjective, clinical assessment based on patient behavior. The combined use of these tools allows for a more complete picture of a patient’s sedation level, potentially enhancing the accuracy of assessment and the effectiveness of sedation management. These values were systematically recorded at two timepoints: 3 and 7 days post-intubation (17–21).

Sevoflurane administration was initiated using an Sedaconda ACD^®^ device at an infusion rate of 6 ml/h, and was adjusted as necessary to achieve the desired clinical effect. The corresponding end-tidal concentrations of sevoflurane were measured using a Vamos^®^ gas analyzer (Dräger, Germany), an anesthetic gas monitor suitable for adult, pediatric, and neonatal patients.

For sedation, a combined approach using propofol and midazolam was adopted. Propofol was initiated at a dose of 3 mg/kg/h, while midazolam was started at 1 mcg/kg/min. Dosages were titrated based on processed EEG monitoring to ensure optimal sedation levels. In cases where posology adjustments were required, emphasis was placed on maintaining midazolam infusion at the lowest possible rate to mitigate its known adverse effects on critically ill patients. The decision to combine propofol and midazolam was influenced by the need for deep sedation in the study cohort. Alongside this sedative regimen, remifentanil was administered at a rate of 0.1 mcg/kg/min to guarantee an adequate level of analgesia.

All patients in both the SEVO and IV groups received standardized treatments for SARS-COV2. The treatment regimen included:

Dexamethasone at a dose of 6 mg/day iv.
IV Infusion of unfractioned heparin (UFH) with the aim to maintain the activated prothrombin time above 60 s.
Paralyzing agents were administered when cycles of pronosupination were deemed necessary.
There were no differences in the treatments for SARS-COV2 between the two groups, ensuring consistency in the management approach across the study population.
All data were recorded in an electronic case report form (e-CRF).
The primary outcome was ICU mortality rate in patients who received inhalatory sedation compared to those received intravenous sedation. The secondary outcomes were examination of potential disparities between the two groups in terms of oxygenation (represented by the PaO2/FiO2 ratio), pulmonary compliance, and levels of D-Dimer, CRP, and creatinine Endpoint.
The primary endpoint was to determine whether patients who received sevoflurane have a lower mortality rate compared to those who received intravenous sedation.
The secondary endpoint was to investigate any differences in PaO2/FiO2 ratio, pulmonary compliance and in D-Dimer, CRP, and creatinine levels between the two groups.

### Bias

In order to minimize potential biases and systematic errors, data collection was conducted by qualified observers who were trained in accordance with Good Clinical Practice (GCP) guidelines. These observers were not involved in the direct clinical care of the patients, thereby reducing the risk of bias in data collection. The data entered into the electronic Case Report Form (e-CRF) was meticulously reviewed and validated by experienced staff to ensure accuracy and consistency. Furthermore, we established clear and objective selection criteria for patient inclusion in the study. This was done to minimize the risk of drop-out and ensure a representative sample for the study.

### Data collection

From August 2021, after approval by the Ethics Committee, all the data listed above were extracted from the medical records and the discharge sheets of the patients enrolled in the study. These data were entered anonymously on the electronic data collection form provided.

### Statistical analysis

The data collected were summarized using the most appropriate descriptive statistics: quantitative variables were summarized using the mean and standard deviation (SD) or the median and interquartile range according to the nature of the variable; for categorical variables, absolute and percentage frequencies were reported.

Missing data were addressed using an imputation method. However, we acknowledge that using the average of the observed values for imputation could potentially introduce bias if the data are not missing at random. Therefore, we carefully evaluated the patterns of missing data to ensure that this method was appropriate and would not significantly distort the results of the study. The Mann–Whitney/Wilcoxon test or *t*-test was used to analyze continuous variables; for categorical variables, Fisher’s exact test or chi-square test was used. Values with *p* < 0.05 were considered significant.

To balance the data from the two enrollment groups (sevoflurane vs. non-sevoflurane) and thus make them comparable in the absence of randomization, a logistic regression model according to propensity score matching (PSM) was applied. The calculation of PSM was performed considering anthropometric data such as age, sex, body mass index (BMI) and comorbidities such as arterial hypertension, chronic obstructive pulmonary disease (COPD), ischemic heart disease, and chronic kidney damage as covariates. All patients were paired using the nearest neighbor without replacement method with a 0.2 caliber and a 1:1 ratio of SEVO to IV.

For primary endpoint, a logistic regression model was applied by setting the mortality rate as the dependent variable and sevoflurane as the independent variable.

All analyses were carried out using the statistical software R (Foundation for Statistical Computing, Vienna, Austria).

## Results

In the period between March 2020 and June 2021, 366 patients were admitted to the COVID intensive care unit of the Santo Stefano Hospital in Prato, Italy. Out of these patients, 265 received invasive mechanical ventilation, and 11 were excluded due to a lack of data at T1, as they were patients transferred from other hospitals. The total number of patients enrolled was therefore 254, out of which 56 were treated with sevoflurane (Group 1) and 198 received intravenous sedation.

**FIGURE 2:**
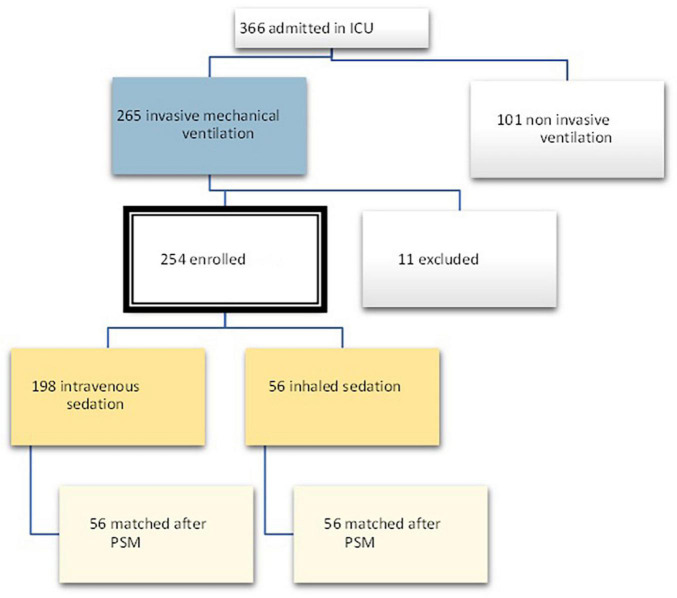
Flow chart for defining the study population. PSM, propensity score matching.

**FIGURE 3:**
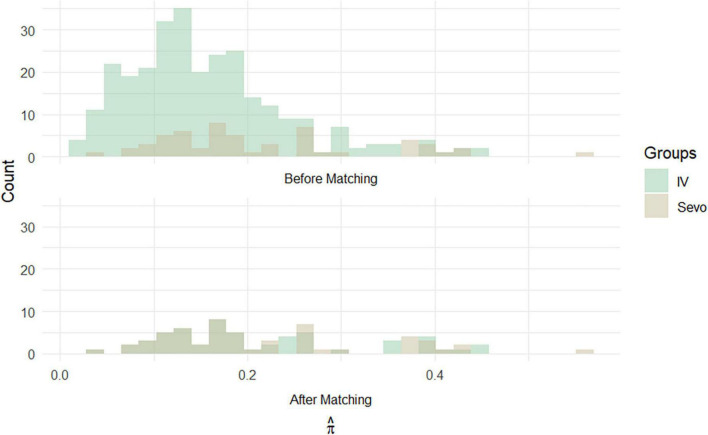
Histograms of propensity score frequencies before and after population matching according to propensity score matching.

On the other hand, the propensity score matching model allowed to select the 56 patients in the IV group by matching them according to the covariates previously selected to those treated with inhalation anesthetic. These 56 patients represented Group 2, i.e., non-sevoflurane population. The final population of the study was therefore composed of a total of 112 patients equally divided into the sevoflurane population (*n* = 56) and the non-sevoflurane population (*n* = 56) (Figures 2, 3).

The characteristics of the enrolled patients after PSM are shown in Table 1 below.

**TABLE 1:** Demographic and clinical course characteristics before admission to the ICU.

	SEVO (Mean ± SD)	IV (Mean ± SD)	*P*-value
Age (year)	63.82 ± 10.70	63.91 ± 13.11	0.9686
BMI (Kg/m^2)	29.99 ± 6.20	29.08 ± 5.25	0.4031
Time Symptoms-Hospitalization (days)	5.35 ± 5.03	4.89 ± 2.96	0.5627
Duration of NIV (days)	3.08 ± 3.23	4.35 ± 4.13	0.08707
Length of stay before ICU (days)	3.25 ± 6.61	4.11 ± 4.56	0.4267
Time to onset of SARS-COV2 after ICU admission (days)	8.72 ± 8.62	9.00 ± 5.00	0.8375
Total History (days)	40.33 ± 21.79	33.25 ± 22.13	0.09367

BMI, body mass index; NIV, non-invasive ventilation; MV, mechanical ventilation.

### Primary endpoint

**FIGURE 4:**
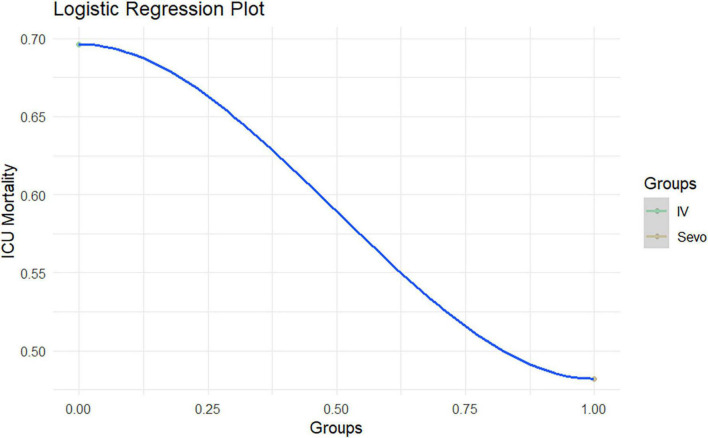
Logistic regression model by ICU Mortality after PSM.

A logistic regression model was applied to the paired sample, relating the outcome (dependent variable) to the treatment used (independent variable). The model showed a beta value of **−**0.9 with a *p*-value = 0.02 valid to reject the null hypothesis (Figure 4). The odds ratio is 0.40 (0.18–0.87).

### Respiratory and ventilatory parameters

**FIGURE 5:**
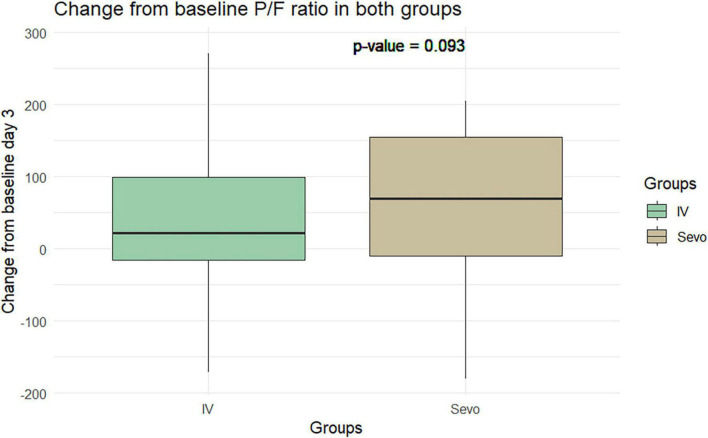
Change in P/F in the two groups of patients after 3 days of mechanical ventilation.

**FIGURE 6:**
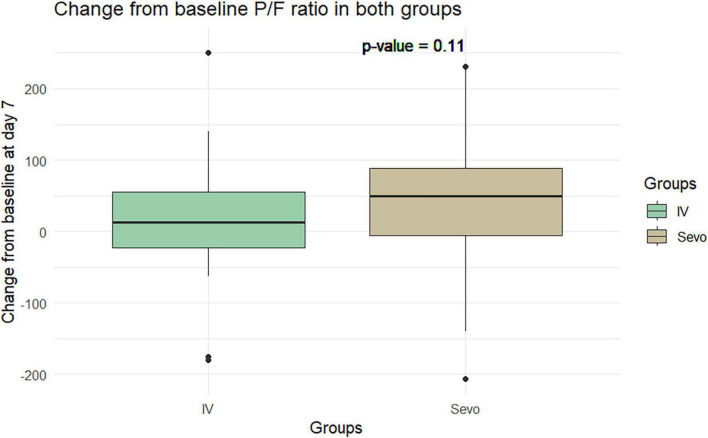
Change in P/F in the two groups of patients after 7 days of invasive mechanical ventilation.

The Wilcoxon test application revealed an improvement in the P/F ratio in the sevoflurane group compared to the IV group at T2 relative to T1. The improvement was 60.8 ± 97.5 in the sevoflurane group and 34.28 ± 90.59 in the IV group. However, this difference was not statistically significant (*p* = 0.06). This pattern was observed again on the seventh day of ventilation, where the P/F ratio improved to 34 ± 98.4 in the sevoflurane group and 9.91 ± 80.37 in the Group 2. Despite this improvement, the difference was not statistically significant, with a *p*-value of 0.10 (Figures 5, 6).

In sevoflurane group there was a statistically significant increase of static compliance (2.37 ± 6.56 cmH_2_O/mL vs. **−**1.83 ± 9.48 in IV) with a *p*-value = 0.02. However this finding was not replicated at T3 (**−**1.57 ± 9.47 in SEVO vs. **−**5.43 ± 9.62 in IV cmH_2_O/mL) with a *p*-value = 0.10. The Positive End-Expiratory Pressure (PEEP) set at the ventilator differed between the two groups at 3 (**−**0.66 ± 1.58 vs. **−**0.18 ± 1.03 cmH_2_O) and 7 days (**−**0.96 ± 2.57 vs. **−**0.67 ± 1.38 cmH_2_O, but these differences were not statistically significant (*p* = 0.95 and 0.93, respectively).

In the subset of patients who survived, there were no statistically significant differences in the duration of mechanical ventilation (27.32 ± 23.91 vs. 34 ± 22.37 days) (*p*-value = 0.07).

### Sedation

At 3 days after orotracheal intubation, the mean BIS index was 43.4 ± 5.6 for the sevoflurane group and 44.5 ± 6.7 for the IV group. The mean end-tidal sevoflurane recorded was 1.45% at 3 days. Analysis of the data showed no statistically significant differences in BIS index values between the two groups of patients at 3 days (*p*-value = 0.09) and at 7 days (*p*-value = 0.12).

### Ventilation and tracheostomy procedures

All patients in this study received invasive mechanical ventilation. To minimize aerosolization and associated risks, surgical tracheostomies were performed after 6 days of mechanical ventilation. In the sevoflurane group, 50 patients underwent this procedure, whereas 49 patients in the IV group received tracheostomy (Table 2).

**TABLE 2:** Parameters registered in ICU.

	SEVO (Mean ± SD)	IV (Mean ± SD)	*P*-value
Noradrenaline (μg/kg/min)	0.34 ± 0.08	0.36 ± 0.09	0.2793
Rocuronium infusion (days)	5 ± 1	5 ± 1	1
Stage I AKI (count)	7	8	1
PEEP (cmH_2_0)	10 ± 2	10 ± 2	1
Tidal Volume (mL/Kg)	8 ± 1	8 ± 1	1
Mechanical ventilation (days)	23.52 ± 20.24	18.16 ± 16.99	0.1416
Length of Stay in Hospital (days)	34.09 ± 21.64	28.36 ± 22.37	0.171
Extubation failure (count)	7	8	1
Secondary infection (count)	27	27	1
At least one cycle of pronation (count)	56	56	1
Tracheostomy (count)	50	49	1

PEEP, positive end expiratory pressure.

During the 16-h pronation cycles, paralyzing agents were administered to the patients. Rocuronium was the chosen agent, infused at a rate of 0.6 mg/kg/h.

### Blood biochemical parameters

Blood biochemical parameters are shown in Table 3. Regarding the D-dimer values the results showed no statistically significant differences between the two groups compared to the baseline value at both 3 and 7 days (*p*-value = 0.39 and 0.72, respectively). For CRP the results showed that there were no statistically significant differences in the two groups compared to the baseline value at T2 and T3 (*p*-value = 0.22 and 0.72, respectively). For creatinine the results showed that there were no statistically significant differences in the two groups compared with the baseline value at T2 and T3 (*p*-value = 0.15 and 0.38, respectively).

**TABLE 3:** Difference from baseline at T2 and T3 of D-dimer, CRP and creatinine.

	SEVO (Mean ± SD)	IV (Mean ± SD)	*P*-value
DDIMER day 3 (ng/mL)	−4634.1 ± 16636.18	−783 ± 7864.71	0.39
DDIMER day 7 (ng/mL)	−4442.78 ± 17765.02	−3468.62 ± 13000.74	0.72
CRP day 3 (mg/dL)	−0.99 ± 10.93	−1.55 ± 10.28	0.22
CRP day 7 (mg/dL)	0.78 ± 9.79	1.73 ± 12.43	0.73
Creatinine day 3 (mg/dL)	0.13 ± 1.81	−0.13 ± 1.07	0.15
Creatinine day 7 (mg/dL)	−0.04 ± 1.1	0.01 ± 1.21	0.38

CRP, C reactive protein.

We observed the application of sevoflurane to be safe, as no adverse reactions or complications were recorded in patients receiving inhaled anesthetics during the course of this study.

## Discussion

The COVID-19 pandemic has led to a global shortage of intravenous sedatives, prompting the use of volatile anesthetics (VAs) like sevoflurane as alternative sedative agents for critically ill COVID-19 patients. Sevoflurane has shown potential benefits in treating patients with COVID-19 related acute respiratory distress syndrome (C-ARDS) due to its bronchodilatory and anti-inflammatory effects (12, 13, 15, 22–24). However, these effects have not led to a reduction in ICU stay length or mortality.

The most compelling evidence of the potential of VAs to reduce lung damage comes from a randomized controlled trial (RCT) in patients with ARDS, where sevoflurane and midazolam were used for sedation. This study found improved oxygenation and reduced levels of specific cytokines associated with inflammatory lung damage in patients receiving sevoflurane. However, these promising results need to be interpreted with caution, as early improvements in oxygenation may not necessarily translate into better long-term outcomes (22).

The majority of clinical evidence examining the relationship between inhalational sedatives and human lungs comes from one lung ventilation (OLV) studies, limiting the applicability of this data (25). Nevertheless, sevoflurane has shown potential benefits in cardiac surgery, providing protection against ischemia–reperfusion injury (26–28). It has also demonstrated a protective role in cardiac events in coronary bypass patients (28).

In a study of sepsis-induced lung injury aggravated by ventilator-induced lung injury (VILI), sevoflurane administration during mechanical ventilation preserved cellular tight junctions, limiting alveolar exudative overflow (29, 30). Additionally, sevoflurane has shown benefits in patients with ARDS undergoing extracorporeal membrane oxygenation (ECMO) (29).

In this study, a logistic regression model was employed to examine the impact of different treatments on the mortality rate. The results reveal an interesting finding with a statistically significant negative association between the treatment used and the outcome. The beta value of **−**0.9 suggests that the treatment has a considerable effect in reducing mortality. Furthermore, the odds ratio of 0.4 indicates that individuals in the SEVO group had substantially lower odds of experiencing mortality compared to those in the IV group. These findings provide initial evidence to support the potential benefits of using sevoflurane treatment in reducing mortality. However, it is essential to acknowledge the wide 95% confidence interval for the odds ratio, which suggests some uncertainty in the estimate. Further research is needed to validate these results and explore other contributing factors to gain a more comprehensive understanding of the relationship between the treatments and mortality rate. Nonetheless, these findings shed light on the importance of considering treatment options in healthcare settings to improve patient outcomes and warrant further investigation and clinical consideration.

Our study found no statistically significant improvement in oxygenation in patients treated with sevoflurane within the first 72 h after intubation (22). Despite this, our study found higher static compliance values in the sevoflurane group 3 days after intubation (31, 32).

### Strengths

Strengths of our study include the use of propensity score matching (PSM) to limit the effect of confounding factors in observational studies. This method allowed us to compare patients who received propofol and midazolam sedation with those who received sevoflurane during C-ARDS requiring orotracheal intubation in the first 72 h after intubation. Another strength of this study is the inclusion of a *post hoc* sample size calculation. By conducting this calculation based on the alpha error level and considering 112 patients selected after propensity score matching (PSM), the study demonstrated a high statistical power of 0.89. This indicates that the sample size was sufficient to detect meaningful differences between the treatment groups and increases confidence in the study’s findings. The consideration of statistical power enhances the study’s validity and reliability, as it ensures that an adequate number of participants were included to detect significant effects. Overall, this attention to sample size calculation and achieving a desirable level of statistical power strengthens the study’s robustness and enhances the reliability of its conclusions.

### Limitations

Limitations of our study include the retrospective nature of the study. Despite this limitation, our study found a positive association between treatment with sevoflurane and a reduction in overall mortality in treated patients, suggesting a potential protective factor. However, further analysis is needed to identify possible confounding factors and to strengthen these outcome data (33). Increasing the sample size could provide further strength to these outcome data. Due to the retrospective design of our study, we were unable to assess the incidence or impact of delirium and other neurological complications in the patient population.

In summary, this retrospective observational study is one of the first to consider inhalation anesthetic treatment during COVID-19 related acute respiratory distress syndrome (C-ARDS). The use of Propensity Score Matching (PSM) model was a strength of this investigation, helping to mitigate the influence of confounding factors on the statistical analysis results.

Our findings suggest that the use of sevoflurane for deep sedation of intubated and mechanically ventilated C-ARDS patients is feasible and effective. Encouraging results were observed regarding pulmonary compliance values 3 days after initiating sedation therapy with sevoflurane. However, it is important to note that the difference in compliance was not statistically significant, and a difference of 2 cmH_2_0/ml may not be of clinical relevance.

The study also suggests that the use of inhaled sedation in patients with C-ARDS is safe and is not associated with worsening renal function. Although we observed a reduction in mortality in patients exposed to sevoflurane, it’s important to clarify that we did not present a direct comparison between groups with regard to mortality.

## Conclusion

In conclusion, our study supports the feasibility and safety of using sevoflurane as a sedative agent in C-ARDS patients, without demonstrating its inferiority to intravenous sedative agents. These findings, taken together with the known or suspected positive effects of sevoflurane presented in the literature, suggest that physicians can consider using sevoflurane alongside intravenous agents in these patients. However, further studies, particularly multicenter randomized clinical trials, are warranted to confirm the potential of this treatment and better evaluate the effects shown here.

## Data availability statement

The raw data supporting the conclusions of this article will be made available by the authors, without undue reservation.

## Ethics statement

The studies involving humans were approved by the Comitato Etico Area Vasta Centro Regione Toscana. The studies were conducted in accordance with the local legislation and institutional requirements. The participants provided their written informed consent to participate in this study. Written informed consent was obtained from the individual(s) for the publication of any potentially identifiable images or data included in this article.

## Author contributions

GC: Conceptualization, Funding acquisition, Visualization, writing–original draft. IC: Conceptualization, Data curation, Formal Analysis, Investigation, Methodology, Software, writing–original draft, Writing–review and editing. BF: Data curation, Methodology, Validation, Writing–review and editing. LC: Supervision, Validation, Writing–original draft. MP: Supervision, Validation, Writing–original draft. LZ: Supervision, Validation, Writing–review and editing.
